# Embracing the Dark Side: Computational Approaches to Unveil the Functionality of Genes Lacking Biological Annotation in Drug-Induced Liver Injury

**DOI:** 10.3389/fgene.2018.00527

**Published:** 2018-11-20

**Authors:** Terezinha Souza, Panuwat Trairatphisan, Janet Piñero, Laura I. Furlong, Julio Saez-Rodriguez, Jos Kleinjans, Danyel Jennen

**Affiliations:** ^1^Department of Toxicogenomics, GROW School for Oncology and Developmental Biology, Maastricht University, Maastricht, Netherlands; ^2^Joint Research Center for Computational Biomedicine (JRC-COMBINE), Faculty of Medicine, RWTH Aachen University, Aachen, Germany; ^3^Integrative Biomedical Informatics Group, Research Programme on Biomedical Informatics (GRIB), Department of Experimental and Health Sciences (DCEXS), Hospital del Mar Medical Research Institute (IMIM), Universitat Pompeu Fabra, Barcelona, Spain; ^4^European Bioinformatics Institute, European Molecular Biology Laboratory (EMBL-EBI), Cambridge, United Kingdom

**Keywords:** annotation, DILI, gene ontology, text mining, network biology, translational bioinformatics

## Abstract

In toxicogenomics, functional annotation is an important step to gain additional insights into genes with aberrant expression that drive pathophysiological mechanisms. Nevertheless, there exists a gap on annotation of these genes which often hampers the interpretation of results and limits their applicability in translational medicine. In this study, we evaluated the coverage of functional annotations of differentially expressed genes (DEGs) induced by 10 selected compounds from the TG-GATEs database identified as high- or no-risk in causing drug-induced liver injury (most-DILI or no-DILI, respectively) using *in vitro* human data. Functional roles of DEGs not present in the most common biological annotation databases – termed “dark genes” – were unveiled via literature mining and via the identification of shared regulatory transcription factors or signaling pathways. Our results demonstrated that there were approximately 13% of dark genes induced by these compounds *in vitro* and we were able to obtain additional relevant information for up to 76% of those. Using interactome data from several sources, we have uncovered genes such as *LRBA*, and *WDR26* as highly connected in the protein network that play roles in drug response. Genes such as *MALAT1, H19*, and *MIR29C* – whose links to hepatotoxicity have been confirmed – were identified as markers for the most-DILI group and appeared as top hits across all literature-based mining methods. Furthermore, we investigated the potential impact of dark genes on liver toxicity by identifying their rat orthologs in combination with their correlation to drug-induced liver pathologies observed *in vivo* following chemical exposure. We identified a set of important regulatory transcription factors of dark genes for all most-DILI compounds including E2F1 and JUND with supporting evidences in literature and we found *Magee1* correlated with chemically induced bile duct hyperplasia and adverse responses at 29 days in rats *in vivo*. In conclusion, in this study we show the potential role of these poorly annotated genes in mechanisms underlying hepatotoxicity and offer a number of computational approaches that may help to minimize current gaps in gene annotation and highlight their values as potential biomarkers in toxicological studies.

## Introduction

In the field of toxicogenomics, various computational approaches have been developed and upgraded over the years. Nowadays, the most commonly applied method consists of the use of differential analysis, i.e., the application of statistical approaches to identify and biologically annotate differentially expressed genes (DEGs) upon compounds’ perturbation (Khatri et al., 2012; Souza et al., 2016). Genome-wide, unsupervised methods such as gene set enrichment analysis (GSEA), biclustering and weighted co-expression analysis (WGCNA) can be used to identify gene sets associated with specific phenotypes (AbdulHameed et al., 2014; Tawa et al., 2014; Sutherland et al., 2016). Another branch of methods includes network-based analyses such as the clustering of gene sets based on their centrality in molecular networks (Kotlyar et al., 2012), as well as mechanistic modeling in smaller scales such as Boolean logic modeling (Zhang J.D. et al., 2014) and ordinary differential equation (ODE)-based models (Hendrickx et al., 2017)– the latter providing dynamical information of the systems in a more refined granularity.

An important bottleneck across all methodologies, however, is the biological annotation of the gene sets. This biological annotation is provided by collections of pathways or gene sets stored in popular knowledge-driven resources such as Reactome (Fabregat et al., 2018) and the Gene Ontology (The Gene Ontology Consortium, 2017). Despite the ever-increasing amount of information deposited in pathway knowledge databases, gaps on functional protein interaction and other types of biological annotation still exist. In addition, a large number of non-coding genes, i.e., small- and long- non-coding genes and pseudogenes, covering around 37,000 molecular entities whose biological roles elucidation is an ongoing task. The “biological process” branch of the Gene Ontology (GO BP) is one of the most commonly used sources of biological annotations. Nevertheless, GO BP terms only cover 33% (19,691 genes) from the entire human genome (estimated in approximately 60,200 genes according to NCBI’s gene annotation) (Brown et al., 2015). On the pathway side, high-confidence databases such as Reactome comprise only around half of all human protein-coding genes (10,762 genes) (Fabregat et al., 2018) while low-confidence high-coverage databases such as Pathway Commons coverage for coding and non-coding portions of the genome is around 38% (22,754 genes). Furthermore, most common pathway resources only cover information regarding protein coding genes, while the role of non-coding RNAs (ncRNAs) in processes such as disease or drug response, remains uncovered. We argue here that these missing entities should not be neglected due to their potential biological functionality with respect to human health.

Community-based efforts can help to fill this gap. An example of this is the creation of GeneRIF (Mitchell et al., 2003), a platform to share short functional descriptions of genes which are generally observed by experimentalists. Such a database allows users to rapidly scan through the additional functional information on genes of interest which are stored in a standardized format. In parallel, user-friendly text mining tools that allow automatic retrieval of information about gene function from the literature have been developed. One such tool is PubTator (Wei et al., 2013), which supports manual literature curation besides offering a collection of annotated abstracts, including relationships among diseases, genes, and drugs. In addition, even if genes are not annotated for their biological processes, they can still be linked to verified disease signatures with, e.g., DisGeNET (Piñero et al., 2017).

Besides text mining, various emerging computational approaches in Systems Biology have been developed with high potential to be applied for unveiling the functional roles of genes. For instance, the inference of transcription factor (TF) activities based on gene expression data may reflect the common regulatory patterns of signaling pathways which are shared among downstream targets with or without functional annotation (Alvarez et al., 2016; Garcia-Alonso et al., 2018). In parallel, the activity of regulatory signaling pathways can be independently predicted by computational approaches based on the expression of genes that reflect the activities of the respective pathway upon perturbation, thus highlighting possible involvement of signaling modulation via unannotated genes (Tarca et al., 2009; Khatri et al., 2012; Schubert et al., 2018). By investigating the list of genes with unknown function which were applied to derive transcription factors’ activities and signaling pathways’ signatures, one could infer their biological functions associated to the role of the predicted upstream regulatory modules.

Recently, Sutherland et al. (2016, 2017) have shown that gene expression in chemically exposed rats coalesce into groups of co-expressed genes (i.e., modules) – some of which appear to be correlated to phenotypes indicative of toxicity or adverse outcomes. Interestingly, this approach highlighted branches comprising a number of modules of interest with little or no biological annotation, some of which containing ncRNAs. Their roles in cellular functioning and disease are slowly being elucidated (Luo et al., 2016; Xu et al., 2017), but their modulation upon drug exposure remains largely uncovered. In spite of that, toxicologists have pointed that their involvement in apical effects should be investigated and considered in regulatory frameworks, i.e., mode-of-action (MoA) and adverse outcome pathway (AOP) analyses (Aigner et al., 2016). Studies to unveil the functionality of these poorly annotated genes are therefore necessary to generate potentially novel biomarkers to improve risk assessment during the preclinical phase. In addition, connecting the poorly annotated genes to the pathological outcomes of rodent studies will further aid to identify their function. Therefore, the identification of human orthologs is imperative to allow and improve translation of the rodent data to the human context.

Therefore, in this work we aim to assess the coverage of the current functional annotation of genes represented in public databases using toxicogenomics sets; those not found in these representative biological annotation databases were coined “dark genes” in this study. Our second goal is to (a) estimate the relevance for cellular functions of dark genes involved in drug response, and (b) assign putative functions to them. For the first task, we assess the presence of these genes in human interactomes built from several sources, in literature-based resources and their association to diseases. For the second, we employed computational approaches to identify (i) common regulatory transcription factors and (ii) signaling pathways’ signatures which are shared between annotated and unannotated genes. Finally, we examine these chemical-induced changes in the light of toxicity and as potential markers of drug-induced liver injury (DILI) given their regulation in human *in vitro* and associations to pathological responses in rat *in vivo*.

## Materials and Methods

### Compound Selection

In order to obtain robust modulation of genes and minimize noisy expression, we opted for analyzing inducible responses across multiple compounds. To investigate whether gene modulation of entities of interest is associated with distinct toxicities, we created two equally sized groups of chemicals to avoid sample bias, selected according to their current classification as agents involved in human DILI. For this, we used a classification based on weight of evidence of causality (DILIRank) (Chen et al., 2016), which categorize compounds in three main classes: most-DILI (drugs withdrawn or with severe DILI indication), less-DILI (drugs with mild DILI indication or adverse reactions) and no-DILI. Here, we selected compounds available on TG-GATEs either classified as most-DILI (acetaminophen, diclofenac, isoniazid, nimesulide, and valproic acid) and no-DILI (caffeine, chloramphenicol, chlorpheniramine, hydroxyzine, and theophylline) to enable an unambiguous separation of gene modulation responses. Further information on the compounds and classification proposed by Chen et al. (2016) can be found in Supplementary Table S1.

### Gene Expression Data: Processing and Differential Gene Expression

Gene expression data were obtained from TG-GATEs^1^ (Igarashi et al., 2015). Raw data files generated *in vitro* from primary human hepatocytes from each compound selected were processed (quality control, background correction, RMA normalization) using the R package affy (Gautier et al., 2004). Genes were annotated with a customCDF (v. 19) with Entrez gene identifiers for Affymetrix GeneChip Human Genome U133 Plus 2.0 arrays. Here, we opted for a traditional approach (i.e., comparison of treated vs. control mean expression) to obtain DEGs; to obtain maximal transcriptional response, we selected the highest dose and latest time point (24 h) from each compound. Differential expression analysis was then performed on each set using the R package LIMMA and comparing to time-matched controls from each compound treatment. DEGs were selected based on their significance after multiple testing correction (false discovery rate, FDR) and an absolute fold change of 1.5 (equivalent to log2 fold change of 0.585) with FDR < 0.05.

### Coverage of Biological Annotation Across Databases

To compute the number of DEGs that were not included in the most commonly used resources in the field of toxicology and network biology, we downloaded the files from Gene Ontology^2^ (The Gene Ontology Consortium, 2017), Reactome^3^ (Fabregat et al., 2018), MSigDB (Liberzon et al., 2015) curated pathways^4^, Pathway Commons ^5^ (Cerami et al., 2011), and OmniPath^6^ (Türei et al., 2016) on May, 2018.

We mapped the gene symbols to Entrez gene identifiers using the file http://ftp.ncbi.nih.gov/gene/DATA/GENE_INFO/Mammalia/Homo_sapiens.gene_info.gz downloaded on April, 2018. For those genes for which we could not find an Entrez gene identifier, we used the correspondence between UniProt identifiers and Entrez gene identifiers from the file http://ftp.ebi.ac.uk/pub/databases/genenames/new/tsv/hgnc_complete_set.txt downloaded on May, 2018. From the Gene Ontology file, we only took into account the GO BP branch as this branch provides a better insight into the biological mechanisms compared to molecular function (MF) and cellular component (CC). From Pathway Commons, we removed interactions without pathway annotations. From OmniPath, we removed interactions that were supported only by protein-protein interaction databases (BioGRID, HPRD, and IntAct). A DEG was tagged as dark gene if it was absent in the pathway databases and GO BP branch.

Furthermore, to assess the global coverage of the biological annotations, the same steps were performed to categorize all genes measured within the Affymetrix array platform.

### Protein Interaction Networks

We built four protein interaction networks (PINs) using data from the most comprehensive, and updated databases: INBIOMAP (Li et al., 2017), HIPPIE (Alanis-Lobato et al., 2017), BIANA (Garcia-Garcia et al., 2010), and IntAct (Orchard et al., 2014).

To build a HIPPIE-based network, we downloaded the file http://cbdm-01.zdv.uni-mainz.de/∼mschaefer/hippie/hippie_current.txt on January, 2018. In the case of INBIOMAP, we downloaded the file from https://www.intomics.com/inbio/map/#downloads. We removed predicted interactions. To build an interactome from BIANA, we downloaded the *Homo sapiens* data from http://sbi.imim.es/web/GUILDify2.php/downloads on January, 2018. For IntAct, we downloaded the file http://ftp.ebi.ac.uk/pub/databases/intact/current/all.zip on October, 2017.

### Literature-Based Resources

To provide further insight on the relevance of the role of the dark genes, we checked if they were involved in human diseases using DisGeNET data, version 5 (Piñero et al., 2017). Additionally, we assessed the presence of dark genes in the scientific literature. For that goal we used GeneRIF (Mitchell et al., 2003), that describe in a short phrase (less than 25 characters in length) the function or functions of a gene, and PubTator (Wei et al., 2013), a web tool that supports manual literature curation using text-mining techniques.

GeneRIFs were downloaded from http://ftp.ncbi.nih.gov/gene/GeneRIF/generifs_basic.gz and PubTator data was downloaded from http://ftp.ncbi.nlm.nih.gov/pub/lu/PubTator/gene2pubtator.gz and http://ftp.ncbi.nlm.nih.gov/pub/lu/PubTator/bioconcepts2pubtator.gz on January 2018.

### Identification of Common Regulatory Transcription Factors and Signaling Pathways

The list of dark genes was mapped to the list of transcription factors and their regulated genes (“regulons”) from the tool DoRothEA (Garcia-Alonso et al., 2018) and to the list of gene signatures used for the inference of signaling pathways’ activities from the tool PROGENy (Schubert et al., 2018). The mapping was classified and compared according to the group of compounds. The shared common transcription factors and signaling pathways in each group were intersected to derive the most representative proxies which represent the corresponding dark genes. Venn diagrams of these results as the ones from PINs (see section “Protein Interaction Networks”) were generated with the following web tool: http://bioinformatics.psb.ugent.be/webtools/Venn.

### Comparison to Weighted Gene Co-expression Network Analysis (WGCNA) Modules

Co-expression analyses aim to obtain significant relationships among genes showing similar patterns of expression across samples. The resulting gene sets (also known as modules) are useful for reducing dimensionality and correlating molecular changes to an observed phenotype. Since clusters are generated in an unbiased manner, it is possible to identify modules encompassing genes with multiple levels of biological annotation (e.g., GO terms or pathways).

To investigate the relevance of these dark genes in an animal model and its implications in adverse outcomes, we identified rat orthologs of the dark genes present in co-expression modules detected in Sutherland et al. (2017). The rat orthologs to human genes were then mapped to modules identified using the annotation available in the Rat Genome Database (rgd.mcw.edu). From there, modules associated with pathological outcomes and underlying GO BPs were further investigated.

## Results

### Compound-Induced Gene Expression

The number of DEGs modulated by each compound can be found in Table 1. By merging the DEGs groupwise, a total of 5,446 and 3,845 genes were found to be induced by most-DILI and no-DILI groups, respectively, comprising in total 6,918 unique genes. These genes were classified using the Ensembl gene annotation information, which showed that the majority of all genes identified were protein coding (95%), followed by non-coding RNA (ncRNA, 4.2%), pseudogenes, snoRNA and others (less than 1% each). An overview of the number of DEGs shared by compounds from the same DILI risk group can be found in the Supplementary Table S1.

**Table 1:** Number of differentially expressed genes (DEGs, absolute FC > 1.5 and FDR < 0.05) of compounds from most-DILI and no-DILI groups.

Most-DILI	Number of DEGs	No-DILI	Number of DEGs
Acetaminophen	2,280	Caffeine	2,316
Diclofenac	1,888	Chloramphenicol	108
Isoniazid	1,024	Chlorpheniramine	93
Nimesulide	1,697	Hydroxyzine	815
Valproic acid	2,290	Theophylline	2,918
**Total unique DEGs**	**5,446**	**Total unique DEGs**	**3,845**

### Biological Annotation and Gene Annotation of Dark Genes

Among the 6,918 genes deemed significantly affected by chemical exposure, 916 genes (∼13%) were not included in any biological pathway or process. This number is lower than the number of genes in the array lacking this type of annotation, identified as 22% (4,210 out of 19,441 genes). In total, 760 out of 916 entities were categorized into gene types based on Ensembl annotation; the majority of those is considered protein coding (Table 2). A detailed description of gene types from the array and modulated by chemicals can be found in the Supplementary Table S1. A comparison of database coverage can be found in Supplementary Date Sheet S1. In addition, a comprehensive list encompassing gene modulation per compound/DILI risk group, as well as pathway and GO annotation and results from the methodologies applied for annotation of the dark genes is available as Supplementary Table S1 while an overview of gene modulation shared across compounds from each group is available in Supplementary Data Sheet S1.

**Table 2:** Classification of genes without GO BP annotation and absent on Reactome, MSigDB, OmniPath, and Pathway Commons databases (dark genes) modulated by compounds from most-DILI and no-DILI groups.

Gene type	Array dark genes	Most-DILI	No-DILI	Dark DEGes
Protein coding	1,756	444	278	567
Antisense RNA	527	69	33	78
lincRNA	722	53	33	63
Processed transcript	113	11	8	15
Pseudogenes^1^	56	18	14	25
snoRNA	8	4	3	5
Sense intronic	25	3	1	3
Sense overlapping	10	1	1	2
miRNA	3	1	0	1
TEC^2^	11	1	1	1
**Total**	**3231**	**605**	**372**	**760**

*^1^Pseudogenes from the categories “transcribed unprocessed pseudogene,” “transcribed unitary pseudogene” and “transcribed processed pseudogene.” ^2^TEC: to be experimentally confirmed.*

### Characterization of Dark Genes in the Human Interactome

Furthermore, we investigated the coverage of the dark genes in four different sources of human protein–protein interactions. We found 492, 420, 475, and 285 dark genes included in HIPPIE, IntAct, Inbiomap and Biana interactomes, respectively. Among them, 536 dark genes were present in at least one of these resources, while 268 were included in all four resources. The overlaps can be found in Supplementary Data Sheet S1.

**FIGURE 1:**
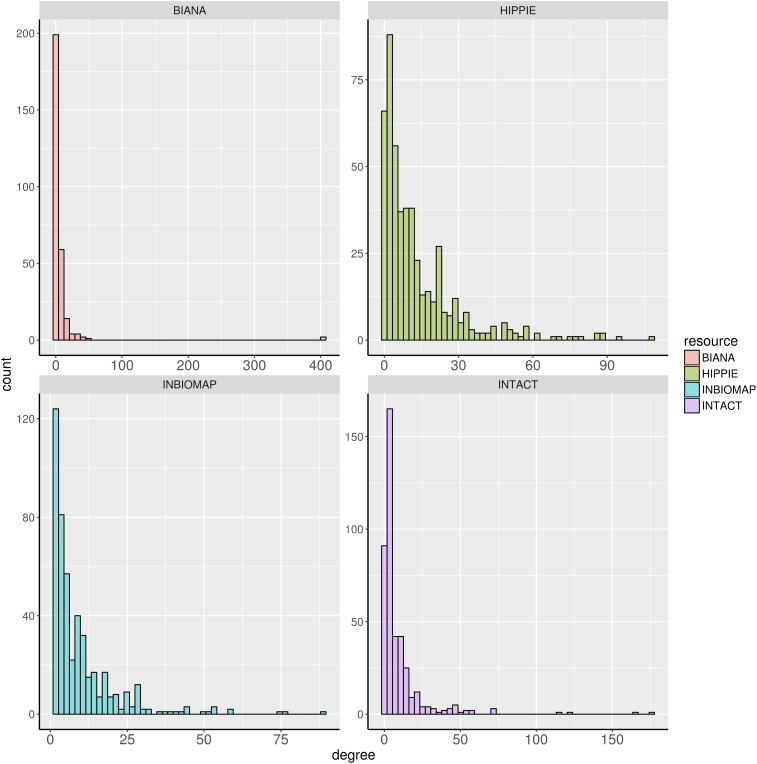
Degree distribution of the dark genes in human interactome databases Biana, HIPPIE, Inbiomap, and IntAct.

We further characterized the dark genes present in the interactomes. Figure 1 shows histograms of the degree distribution of the dark genes in each interactome. A large fraction of the dark genes has low connectivity in all four interactomes, although there are some genes with relatively high degrees. Some examples of these latter genes, more connected than the rest of dark genes in the four interactomes, are shown in Table 3.

**Table 3:** Degree of connectivity for top 10 genes in human protein–protein interaction databases.

Gene symbol	Description	BIANA	HIPPIE	INBIOMAP	IntAct
*RBM12*	RNA binding motif protein 12	405	44	17	8
*LRBA*	LPS responsive beige-like anchor protein	402	28	16	9
*SGTB*	Small glutamine rich tetratricopeptide repeat containing beta	8	87	88	177
*TMEM25*	Transmembrane protein 25	3	85	77	2
*FAM189A2*	Family with sequence similarity 189 member A2	10	78	75	10
*ZCCHC10*	Zinc finger CCHC-type containing 10	51	52	53	59
*C1orf109*	Chromosome 1 open reading frame 109	45	51	53	116
*TSSC4*	Tumor suppressing subtransferable candidate 4	13	68	59	17
*WDR26*	WD repeat domain 26	3	79	51	33
*FAM90A1*	Family with sequence similarity 90 member A1	33	49	50	122

### Literature Mining: Disease Association, GeneRIF, and PubTator

We also evaluated other literature-based resources containing functional information. First, we used DisGeNET v5.0 to determine whether the dark genes are associated to human diseases. We found 60 dark genes with disease annotations reported by curated databases, and 255 dark genes in DisGeNET ALL dataset, which also includes the results from automatic text mining in the scientific literature. The top genes with disease annotations in the curated data in DisGeNET are shown in Table 4. The diseases in which these genes were more frequently involved were different types of neoplasms, although they seem to play a role in a wide variety of diseases, and abnormal phenotypes (Supplementary Table S1).

**Table 4:** Top 10 genes associated to diseases in DisGeNET (curated data).

Symbol	Description	Gene type	DILI risk group(s)	Number of diseases
*CLIP2*	CAP-Gly domain containing linker protein 2	Protein-coding	Most-DILI	141
*IPW*	Imprinted in Prader-Willi syndrome (non-protein coding)	ncRNA	Most-DILI, no-DILI	66
*TGDS*	TDP-glucose 4,6-dehydratase	Protein-coding	Most-DILI	62
*LRBA*	LPS responsive beige-like anchor protein	Protein-coding	Most-DILI	33
*AMMECR1*	Alport syndrome, mental retardation, midface hypoplasia and elliptocytosis chromosomal region gene 1	Protein-coding	Most-DILI, no-DILI	27
*TMEM98*	Transmembrane protein 98	Protein-coding	Most-DILI	9
*H19*	H19, imprinted maternally expressed transcript (non-protein coding)	ncRNA	Most-DILI	7
*MALAT1*	Metastasis associated lung adenocarcinoma transcript 1 (non-protein coding)	ncRNA	Most-DILI	7
*WDR11*	WD repeat domain 11	Protein-coding	Most-DILI	6
*CMYA5*	Cardiomyopathy associated 5	Protein-coding	no-DILI	3

We also evaluated the coverage of the dark genes in GeneRIF which contains users-submitted compact information regarding the function of the genes. We found 356 dark genes with GeneRIF annotations. Twenty-three dark genes had 10 or more GeneRIFs, and among those, several ncRNAs (Table 5). Some relevant examples of the GeneRIFs for *MALAT1* are “*MALAT1* level is associated with liver damage, and has clinical utility for predicting development of hepatocellular carcinoma” or “observations suggest that *MALAT1* promotes hepatic steatosis and insulin resistance by increasing nuclear SREBP-1c protein stability.”

**Table 5:** Top 10 dark genes by number of GeneRIFs with their corresponding number of publications indexed on PubTator.

Symbol	Description	Gene Type	DILI risk group(s)	GeneRIFs	Number of publications
*H19*	H19, imprinted maternally expressed transcript (non-protein coding)	ncRNA	Most-DILI	193	1169
*MALAT1*	Metastasis associated lung adenocarcinoma transcript 1 (non-protein coding)	ncRNA	Most-DILI	156	1203
*MIR29C*	microRNA 29c	ncRNA	Most-DILI	77	234
*UCA1*	Urothelial cancer associated 1 (non-protein coding)	ncRNA	Most-DILI, no-DILI	63	152
*NEAT1*	Nuclear paraspeckle assembly transcript 1 (non-protein coding)	ncRNA	Most-DILI, no-DILI	56	223
*PVT1*	Pvt1 oncogene (non-protein coding)	ncRNA	Most-DILI	56	182
*TUG1*	Taurine up-regulated 1 (non-protein coding)	ncRNA	Most-DILI, no-DILI	41	99
*MTUS1*	Microtubule associated scaffold protein 1	Protein-coding	Most-DILI, no-DILI	26	71
*TM4SF5*	Transmembrane 4 L six family member 5	Protein-coding	Most-DILI, no-DILI	20	37
*FAM167A*	Family with sequence similarity 167 member A	Protein-coding	Most-DILI	19	32

A similar exercise was performed using PubTator to obtain additional information with a unbiased text-mining approach. We found that 550 dark genes matched the entries in PubTator. Interestingly, the two genes with the highest number of hits were, again, two long non-coding RNAs, *MALAT1* and *H19* (Table 5), with over 1,000 papers each. In some cases a single entry on PubTator was a match for multiple hits, as for instance “Central role of the p53 pathway in the non-coding-RNA response to oxidative stress,” which related *MALAT1*, *NEAT1*, and *PVT1* (3 dark ncRNAs) to oxidative stress produced by H2O2 (Fuschi et al., 2017).

### Mapping Functional Information of the Dark Genes With Common Regulatory TF and Signaling Pathways

**FIGURE 2:**
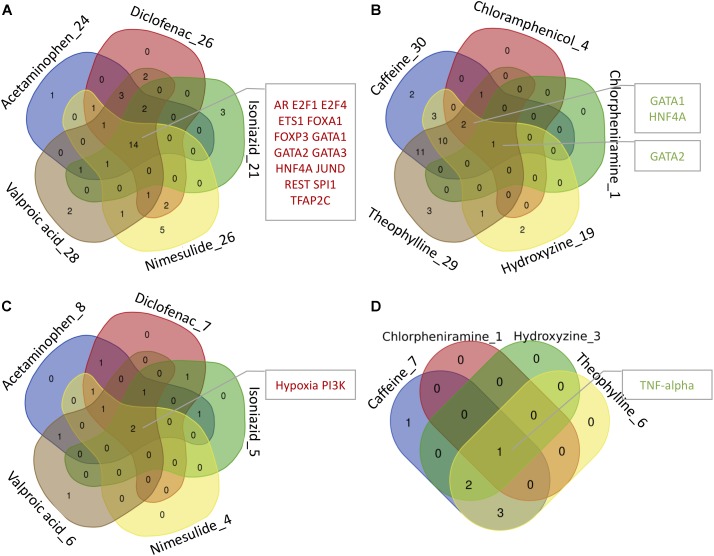
Venn diagrams showing the intersection of transcription factors (TFs) and signaling pathways regulating at least one dark gene. The number accompanying each compound refers to the number of transcription factors and signaling pathways enriched by dark genes and the intersected modules by all or most of the compounds are highlighted in the adjacent boxes. **(A,B)** Regulatory TFs of hepatotoxic and non-hepatotoxic compounds, respectively. **(C,D)** Regulatory signaling pathways of hepatotoxic and non-hepatotoxic compounds, respectively. No enriched signaling pathway was found for Chloramphenicol (absent in **D**).

By mapping the DEGs of the selected compounds, we found that about 16% of dark genes are the targets genes of regulatory TFs in DoRothEA (Table 6). The intersections of regulatory TFs between most-DILI and no-DILI compounds are shown in Figure 2. Here, the most representative TFs for most-DILI group overlapped across all five compounds (*n* = 14) were AR, E2F1, E2F4, ETS1, FOXA1, FOXP3, GATA1, GATA2, GATA3, HNF4A, JUND, REST, SPI1, and TFAP2C, while the most representative for non-DILI group shared by all five compounds (*n* = 1) was GATA2.

**Table 6:** Overview of mapped dark genes based on transcriptional regulation (DoRothEA) and on signaling pathway signatures (PROGENy).

Compound	Dark genes	Dark genes in DoRothEA	Number of mapped TFs	Dark genes in PROGENy	Number of mapped signaling pathways
Acetaminophen	294	46	24	11	8
Valproic acid	330	51	28	11	6
Isoniazid	152	22	21	8	5
Diclofenac	221	32	26	10	7
Nimesulide	145	34	26	5	4
**Total Most-DILI**	732	115	40	29	10
Theophylline	326	48	29	16	6
Caffeine	271	47	30	16	7
Hydroxyzine	81	17	19	3	3
Chloramphenicol	6	2	4	0	0
Chlorpheniramine	7	1	1	1	1
**Total No-DILI**	451	70	36	19	7

In parallel, we found that about 4% of dark genes can be grouped together with the gene signatures used for the inference of signaling pathways’ activities in PROGENy (Table 6). The most representative signaling pathways overlapped among all five most-DILI compounds (*n* = 2) were Hypoxia and PI3K, whereas TNF-alpha was the most representative one for non-DILI compounds (excluding chloramphenicol which did not have an enriched pathway), see Figure 2.

The scripts for all analyses conducted in this study are available in Supplementary Data Sheets S2, S3.

### Rat Orthologs to Human Dark Genes in Co-expression Modules

Identification of rat orthologs to human dark genes and comparison to co-expression modules generated from rats exposed to chemicals showed that 544 human dark genes had an ortholog in rat and, from these, 241 were included in at least one WGCNA module. Among these genes, at least 20 comprised those coding for transmembrane proteins (TMEM family). These dark genes were found in (1) modules from branches with global poor GO BP annotation (branches C.I and C.II indicated by Sutherland et al., 2017) and (2) modules associated with pathology. Table 7 contains a list of dark gene orthologs whose modules were associated with specific pathologies and the underlying GO BP (whenever available). The complete list of dark genes orthologs mapped to modules can be found in Supplementary Table S1.

**Table 7:** Orthologs to human dark genes present in modules associated to pathologies in rats described by Sutherland et al. (2017).

Module	Gene symbol	Pathology association	GO-BP
13m	*Smim14*	Adverse at 29 days, Hematopoiesis	Complement activation; Inflammatory response, Leukocyte chemotaxis
39	*Lhfpl6*	BDH	Extracellular matrix organization, Collagen fibril organization
205	*Thyn1*	BDH, Adverse at 29 days	Cellular response to DNA damage stimulus, Signal transduction by p53 class mediator
293	*Magee1*	BDH	–
55m	*Abracl*	Fibrosis, BDH, Necrosis	Membrane raft assembly, Regulation of cytoskeleton organization
14m	*Wdr70, Lyrm1, Tmem209*	Hypertrophy	Protein folding, tRNA metabolic process
10	*RGD1560010, Abhd8, Tbc1d31*	Increased mitosis	Cell cycle, Mitotic cell cycle
81	*Jpt1*	Increased mitosis, BDH	Actin polymerization or depolymerization
70	*Spata2l, Ubald1*	Single cell necrosis	Cell cycle arrest
309	*RGD1359127*	Single cell necrosis	–
147	*Oser1*	Single cell necrosis	–
27m	*C2cd2*	Vacuolation	

*BDH, bile duct hyperplasia.*

## Discussion

Pathway and network analyses are essential steps downstream to the identification of interesting features (e.g., differential analysis) in diverse fields of ‘omics research. Despite advances in biological annotation of the human genome, there is still a considerable gap in knowledge, owed mainly to experimental evaluation of already well-studied entities, which hampers biomedical research (Haynes et al., 2018). In this study, we aimed to investigate these poorly annotated entities (coined dark genes) in the light of chemical exposure since many studies in mechanistic toxicology are heavily attached to biological roles and many genes with potential mechanistic and predictive roles may remain uncovered as a result.

From our analysis, we observed that approximately 13% of DEGs and 22% of all genes in the array were not mapped to GO BP, OmniPath, MSigDB, Reactome or Pathway Commons. This finding highlights that the issue with unannotated genes is generalized and the biological functions of a number of DEGs identified in gene expression studies remain to be uncovered. Genes with Ensembl classification were mostly categorized as protein coding (73%), while 8% of dark genes were classified as long-intergenic non-coding RNA (lincRNAs), which have increasing evidences to play a role in drug-induced organ toxicity (Zhou et al., 2015; Dempsey and Cui, 2017).

It was demonstrated that up to 59% of dark genes are present in at least one of the human interactome databases. Of these, a few have higher degree of connectivities to the other genes as shown in Table 3. In the context of drug development, PINs have been employed to understand the perturbations elicited by drug treatment in cellular processes, and to characterize drug targets (Yıldırım et al., 2007) and side effects (Wang et al., 2013). Recently, Piñero et al. (2018) has shown that within the set of drug targets, those that are related to side effects are more central in the interactome at local, global and meso-scale level. In the current study, we have used interactome data to highlight genes with strong molecular data, such as genes *LRBA*, which showed over 400 interaction partners in BIANA database, being associated to several diseases and involved in the response to DNA damage (Matsuoka et al., 2007). Another example is *WDR26* – with over 70 partners in HIPPIE database and also disease-associated, that has been found to protect cells from oxidative stress-induced apoptosis (Zhao et al., 2009). Furthermore, genes such as *MYO15B, BEX5, C12orf75*, and *SPATA2L*, that appear differentially expressed in at least 4 of the 5 DILI compounds and not perturbed upon no-DILI drugs, are also involved in protein-protein interactions according to most PPI databases, thus making them interesting potential DILI biomarker candidates to further pursue.

**FIGURE 3:**
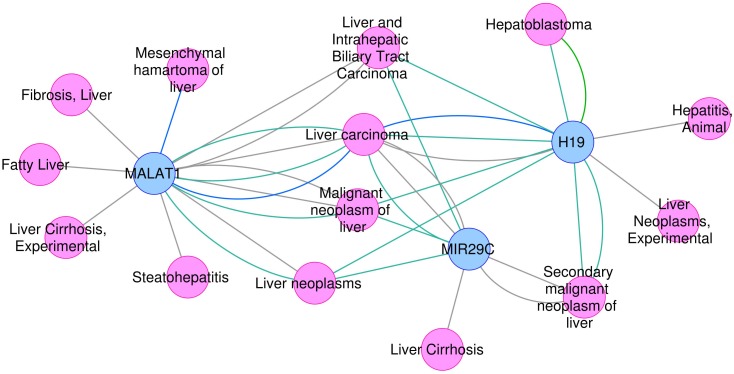
Association between *H19*, *MALAT1*, and *MIR29C* and liver -related disease phenotypes.

On the other hand, the use of text mining tools allowed to obtain information about non-coding RNAs – entities which are not included in PINs. With these methods we identified genes such as microRNA MIR29C, and non-coding RNAs H19 and MALAT1, all found exclusively in the most-DILI risk group. Deregulation of *H19* and *MALAT1* has been associated with liver disease (Takahashi et al., 2014). Downregulation of *H19*, which was consistently observed in all most-DILI compounds except nimesulide, has been associated with formation of Mallory-Denk bodies (MDBs), aggresomes of proteins found in many types of liver diseases (Oliva et al., 2009). Furthermore, downregulation of circulating microRNAs from the mir29 family were shown in liver cirrhosis patients (Loosen et al., 2017) and *MIR29C* in particular has been associated to acute and chronic models of hepatotoxicity (Schueller et al., 2018). The relevance of these genes in diseases, in particular liver diseases, was demonstrated in the disease association analysis with DisGeNET (Figure 3). Clear associations to common compound-induced liver injuries (fatty liver, fibrosis, steatohepatitis, and cirrhosis), in addition to cancer-related processes, were observed. Drug-disease relationships are regarded as important ways to improve toxicity testing and drug safety and discovery; methods such as Connectivity map have been successfully applied to datasets, showing that correlation of ‘omics’ profiles between certain drugs and disease profiles recapitulate drug disease risks (Lamb et al., 2006; Caiment et al., 2014). Here, we show the potential of poorly annotated genes to strengthen these connections, impacting the discovery of potentially novel toxicity markers.

On another perspective, even though regulatory TF and pathway enrichment analyses have already been widely applied to many fields in biomedicine especially in cancer research (Darnell, 2002; Bhagwat and Vakoc, 2015), only a few case studies were shown in the field of drug safety and toxicity (Souza et al., 2017). Our unbiased enrichment analysis of regulatory TFs and pathways is one of the first studies to combine the analysis of both transcription factors and signaling pathways related to drug toxicity, especially focusing on poorly annotated entities regulated by these systems in an effort to propose additional markers of drug toxicity (Andersen et al., 2013; Jennings et al., 2013).

In our analyses we show that approximately 16% of the dark genes were mapped in TF-regulon database DoRothEA (Table 6). Among the enriched TFs of dark genes in the most-DILI group, we detected, for instance, E2F1, which has been demonstrated to be involved in liver fibrosis, a common end-point of compound-induced liver injury (Zhang Y. et al., 2014), as well as JUND in the inflammatory process in liver (Seki et al., 2012). Pathways’ signatures, which are largely curated and expected to represent the activity states of signaling pathways, were also found to contain approximately 4% of dark genes modulated in this study. Enriched pathways for these entities included the Hypoxia pathway, known to play a role in inflammation and fibrosis (Nath and Szabo, 2012), PI3K pathway, that mediates liver injury in chronic fluorosis (Fan et al., 2015), as well as that of TNF-alpha pathway as the mediator of hepatotoxicity and regeneration (Schwabe and Brenner, 2006), inflammation and homeostasis (Tacke et al., 2009). These liver-injury mediating TFs E2F1 and JUND together with the representatives from hepatotoxic-related pathways such as HIF1A, AKT, and TNF-receptor could be perceived as potential markers to demonstrate the involvement of the dark genes in the context of DILI.

By comparing the dark genes identified in human hepatocytes to corresponding orthologs *in vivo* in a murine model, we found a consistency in the expression of these entities across species. More importantly, we show that these genes are associated with pathological outcomes (Table 7), highlighting their potential value in pre-clinical studies. We were not able to assess the relevance of aforementioned genes linked to DILI (*MALAT1*, *H19*, and *MIR29C*) since these genes, although possessing rat orthologs, were not measured in the arrays. However, functional annotation performed *in vitro* pointed similarities to most-DILI risk – demonstrated through genes such as *Magee1*. *Magee1* was modulated *in vitro* only by compounds in the most-DILI group, and associated to “Liver Cirrhosis, Experimental” according to DisGeNET data; *in vivo*, it was found in a module associated with hepatobiliary outcomes (Sutherland et al., 2017). Furthermore, genes such as *Smim14* and *Thyn1* were included in modules with biological processes; these functions may be putatively associated to these genes, as it has been shown that genes acting simultaneously often share the same biological process(es), and therefore gene co-expression networks can be used for the purpose of functional annotation (van Dam et al., 2017).

By combining the results of all approaches employed in this study, we were able to find evidence in at least one approach for 701 out of the initial 916 dark genes, i.e., 76% (Supplementary Table S1). Some genes were consistently found across all methodologies in addition to rat-human orthologs mapped to co-expression modules (e.g., *ST7, KLHDC2, CCDC28A, TMEM140, TRIM47*), all of which were included in clusters with GO BP annotation (Supplementary Table S1) (Sutherland et al., 2017). Genes exclusively modulated by the most-DILI group with (i) hits across several methods (i.e., sum of evidences equal or higher to 8, see Supplementary Table S1) (e.g., *ST7, LRBA, TPD52L2, TSSC4, BOLA1, YIPF1, TMEM168, RSRC2, CCDC92, ITFG1, ZMYND19, TTC14*, and *TMEM9*) and (ii) moderate amount of evidence (sum equal or higher than 4) and associated with pathologies (*MAGEE1*, *TBC1D31*, *SPATA2L*, *ABHD8*, and *LHFPL6*) were also identified. Although there are reports on their involvement in different liver diseases, including non-alcoholic steatohepatitis and hepatocellular carcinoma (Cai et al., 2018; Zhu et al., 2018), their roles in drug-induced organ injury has not yet been investigated. In addition to that, 215 dark genes modulated by the chemicals investigated here remain obscure – the majority (174) being classified as ncRNAs – which have been presented as potential non-invasive disease biomarkers (Teng and Ghoshal, 2015; de Gonzalo-Calvo et al., 2018). Regardless the level of findings, our results indicate concordance *in silico*, *in vitro*, and *in vivo* and potential roles in toxicity that should pave the way for further investigations aiming at the confirmation and uncovering of their biological function.

Overall, our study indicated how limitations arising from the biological annotation of genes can be minimized using a number of computational approaches, especially in the field of toxicogenomics in which uncovering and understanding of drug-gene responses is necessary to obtain novel/robust markers of toxicity. Although comprehensive databases such as Harmonizome (Rouillard et al., 2016) exist, they do not offer advanced mapping into the TF and pathway signatures nor cross-species concordance as performed in this study. It should also be noted that this study was based on a predefined set of approximately 19,000 genes; analyses of data from unconstrained methods (e.g., RNA-seq) using the methods described here will likely be able to provide a more accurate picture of the state of functional annotation of the whole human genome and shed light onto new, potentially relevant features in toxicological analysis.

## Conclusion

In summary, this study highlighted a gap in functional gene annotation in the field of toxicogenomics and presented potential methods that can generate a pipeline to fill such gap through mapping using several resources. We showed that text mining tools and biocuration offer important insights by revealing potential chemical-disease associations and functional roles. The presented microRNA, ncRNAs and regulatory transcription factors in this study may also be further investigated as potential biomarkers of DILI. Nevertheless, further experimental validation of their biological roles are still necessary not only to extend the biological knowledge beyond the scope of well-annotated entities, but in order to also fully understand their roles in toxicity and disease development which would help to unlock their prognostic and translational value.

## Author Contributions

TS performed microarray analysis, cross-species comparison, and module enrichment. PT performed the annotations with transcription factor regulation and signaling pathways’ signatures. JP characterized the genes and performed functional analysis. TS, PT, JP, LF, JS-R, JK, and DJ designed and revised the analyses. TS, PT, and JP wrote the manuscript. All authors read and revised the manuscript.

## Conflict of Interest Statement

The authors declare that the research was conducted in the absence of any commercial or financial relationships that could be construed as a potential conflict of interest.

## Footnotes

1 http://toxico.nibiohn.go.jp/english/datalist.html

2 http://geneontology.org/gene-associations/goa_human.gaf.gz

3 https://reactome.org/download/current/NCBI2Reactome_All_Levels.txt

4 http://software.broadinstitute.org/gsea/msigdb/collections.jsp#C2

5 http://www.pathwaycommons.org/archives/PC2/v10/PathwayCommons10.All.hgnc.txt.gz

6 http://omnipathdb.org/interactions/?fields=sources&fields=references

## Supplementary Material

TABLE S1 Comprehensive overview of the dark genes analyzed, including (i) modulation by individual chemicals and per DILI risk, (ii) current mapping status to gene ontology (GO) and pathways (several databases), and (iii) results obtained from different methodologies applied.

DATA SHEET S1 Supplementary Figures.

DATA SHEETS S2 and S3 Scripts (.R) used in all analyses conducted in this study as well as mapping to databases and IDs.

## References

[B1] AbdulHameed M. D. M. Tawa G. J. Kumar K. Ippolito D. L. Lewis J. A. Stallings J. D. (2014). Systems level analysis and identification of pathways and networks associated with liver fibrosis. *PLoS One* 9:e112193. 10.1371/journal.pone.0112193 25380136 PMC4224449

[B2] Aigner A. Buesen R. Gant T. Gooderham N. Greim H. Hackermüller J. (2016). Advancing the use of noncoding RNA in regulatory toxicology: report of an ECETOC workshop. *Regul. Toxicol. Pharmacol.* 82 127–139. 10.1016/J.YRTPH.2016.09.018 27663666

[B3] Alanis-Lobato G. Andrade-Navarro M. A. Schaefer M. H. (2017). HIPPIE v2.0: enhancing meaningfulness and reliability of protein–protein interaction networks. *Nucleic Acids Res.* 45 D408–D414. 10.1093/nar/gkw985 27794551 PMC5210659

[B4] Alvarez M. J. Shen Y. Giorgi F. M. Lachmann A. Ding B. B. Ye B. H. (2016). Functional characterization of somatic mutations in cancer using network-based inference of protein activity. *Nat. Genet.* 48 838–847. 10.1038/ng.3593 27322546 PMC5040167

[B5] Andersen M. E. McMullen P. D. Bhattacharya S. (2013). Toxicogenomics for transcription factor-governed molecular pathways: moving on to roles beyond classification and prediction. *Arch. Toxicol.* 87 7–11. 10.1007/s00204-012-0980-6 23184225

[B6] Bhagwat A. S. Vakoc C. R. (2015). Targeting transcription factors in cancer. *Trends Cancer* 1 53–65. 10.1016/j.trecan.2015.07.001 26645049 PMC4669894

[B7] Brown G. R. Hem V. Katz K. S. Ovetsky M. Wallin C. Ermolaeva O. (2015). Gene: a gene-centered information resource at NCBI. *Nucleic Acids Res.* 43 D36–D42. 10.1093/nar/gku1055 25355515 PMC4383897

[B8] Cai J. Zhang X.-J. Li H. (2018). Progress and challenges in the prevention and control of nonalcoholic fatty liver disease. *Med. Res. Rev.* 10.1002/med.21515 [Epub ahead of print]. 29846945

[B9] Caiment F. Tsamou M. Jennen D. Kleinjans J. (2014). Assessing compound carcinogenicity *in vitro* using connectivity mapping. *Carcinogenesis* 35 201–207. 10.1093/carcin/bgt278 23940306

[B10] Cerami E. G. Gross B. E. Demir E. Rodchenkov I. Babur O. Anwar N. (2011). Pathway Commons, a web resource for biological pathway data. *Nucleic Acids Res.* 39 D685–D690. 10.1093/nar/gkq1039 21071392 PMC3013659

[B11] Chen M. Suzuki A. Thakkar S. Yu K. Hu C. Tong W. (2016). DILIrank: the largest reference drug list ranked by the risk for developing drug-induced liver injury in humans. *Drug Discov. Today* 21 648–653. 10.1016/j.drudis.2016.02.015 26948801

[B12] Darnell J. E. (2002). Transcription factors as targets for cancer therapy. *Nat. Rev. Cancer* 2 740–749. 10.1038/nrc906 12360277

[B13] de Gonzalo-Calvo D. Vea A. Bär C. Fiedler J. Couch L. S. Brotons C. (2018). Circulating non-coding RNAs in biomarker-guided cardiovascular therapy: a novel tool for personalized medicine? *Eur. Heart J.* 10.1093/eurheartj/ehy234 [Epub ahead of print]. 29688487 PMC6528150

[B14] Dempsey J. L. Cui J. Y. (2017). Long non-coding RNAs: a novel paradigm for toxicology. *Toxicol. Sci.* 155 3–21. 10.1093/toxsci/kfw203 27864543 PMC5216656

[B15] Fabregat A. Jupe S. Matthews L. Sidiropoulos K. Gillespie M. Garapati P. (2018). The reactome pathway knowledgebase. *Nucleic Acids Res.* 46 D649–D655. 10.1093/nar/gkx1132 29145629 PMC5753187

[B16] Fan B. Yu Y. Zhang Y. (2015). PI3K-Akt1 expression and its significance in liver tissues with chronic fluorosis. *Int. J. Clin. Exp. Pathol.* 8 1226–1236. 25973007 PMC4396260

[B17] Fuschi P. Carrara M. Voellenkle C. Garcia-Manteiga J. M. Righini P. Maimone B. (2017). Central role of the p53 pathway in the noncoding-RNA response to oxidative stress. *Aging* 9 2559–2586. 10.18632/aging.101341 29242407 PMC5764393

[B18] Garcia-Alonso L. Iorio F. Matchan A. Fonseca N. Jaaks P. Peat G. (2018). Transcription factor activities enhance markers of drug sensitivity in cancer. *Cancer Res.* 78 769–780. 10.1158/0008-5472.CAN-17-1679 29229604 PMC6522379

[B19] Garcia-Garcia J. Guney E. Aragues R. Planas-Iglesias J. Oliva B. (2010). Biana: a software framework for compiling biological interactions and analyzing networks. *BMC Bioinformatics* 11:56. 10.1186/1471-2105-11-56 20105306 PMC3098100

[B20] Gautier L. Cope L. Bolstad B. M. Irizarry R. A. (2004). affy–analysis of Affymetrix GeneChip data at the probe level. *Bioinformatics* 20 307–315. 10.1093/bioinformatics/btg405 14960456

[B21] Haynes W. A. Tomczak A. Khatri P. (2018). Gene annotation bias impedes biomedical research. *Sci. Rep.* 8:1362. 10.1038/s41598-018-19333-x 29358745 PMC5778030

[B22] Hendrickx D. M. Souza T. Jennen D. G. J. Kleinjans J. C. S. (2017). DTNI: a novel toxicogenomics data analysis tool for identifying the molecular mechanisms underlying the adverse effects of toxic compounds. *Arch. Toxicol.* 91 2343–2352. 10.1007/s00204-016-1922-5 28032149 PMC5429357

[B23] Igarashi Y. Nakatsu N. Yamashita T. Ono A. Ohno Y. Urushidani T. (2015). Open TG-GATEs: a large-scale toxicogenomics database. *Nucleic Acids Res.* 43 D921–D927. 10.1093/nar/gku955 25313160 PMC4384023

[B24] Jennings P. Limonciel A. Felice L. Leonard M. O. (2013). An overview of transcriptional regulation in response to toxicological insult. *Arch. Toxicol.* 87 49–72. 10.1007/s00204-012-0919-y 22926699

[B25] Khatri P. Sirota M. Butte A. J. (2012). Ten years of pathway analysis: current approaches and outstanding challenges. *PLoS Comput. Biol.* 8:e1002375. 10.1371/journal.pcbi.1002375 22383865 PMC3285573

[B26] Kotlyar M. Fortney K. Jurisica I. (2012). Network-based characterization of drug-regulated genes, drug targets, and toxicity. *Methods* 57 499–507. 10.1016/J.YMETH.2012.06.003 22749929

[B27] Lamb J. Crawford E. D. Peck D. Modell J. W. Blat I. C. Wrobel M. J. (2006). The Connectivity Map: using gene-expression signatures to connect small molecules, genes, and disease. *Science* 313 1929–1935. 10.1126/science.1132939 17008526

[B28] Li T. Wernersson R. Hansen R. B. Horn H. Mercer J. Slodkowicz G. (2017). A scored human protein–protein interaction network to catalyze genomic interpretation. *Nat. Methods* 14 61–64. 10.1038/nmeth.4083 27892958 PMC5839635

[B29] Liberzon A. Birger C. Thorvaldsdóttir H. Ghandi M. Mesirov J. P. Tamayo P. (2015). The molecular signatures database hallmark gene set collection. *Cell Syst.* 1 417–425. 10.1016/j.cels.2015.12.004 26771021 PMC4707969

[B30] Loosen S. H. Schueller F. Trautwein C. Roy S. Roderburg C. (2017). Role of circulating microRNAs in liver diseases. *World J. Hepatol.* 9 586–594. 10.4254/wjh.v9.i12.586 28515844 PMC5411953

[B31] Luo F. Liu X. Ling M. Lu L. Shi L. Lu X. (2016). The lncRNA MALAT1, acting through HIF-1α stabilization, enhances arsenite-induced glycolysis in human hepatic L-02 cells. *Biochim. Biophys. Acta Mol. Basis Dis.* 1862 1685–1695. 10.1016/J.BBADIS.2016.06.004 27287256

[B32] Matsuoka S. Ballif B. A. Smogorzewska A. McDonald E. R. Hurov K. E. Luo J. (2007). ATM and ATR substrate analysis reveals extensive protein networks responsive to DNA damage. *Science* 316 1160–1166. 10.1126/science.1140321 17525332

[B33] Mitchell J. A. Aronson A. R. Mork J. G. Folk L. C. Humphrey S. M. Ward J. M. (2003). Gene indexing: characterization and analysis of NLM’s GeneRIFs. *AMIA Annu. Symp. Proc.* 2003 460–464. PMC1480312 14728215

[B34] Nath B. Szabo G. (2012). Hypoxia and hypoxia inducible factors: diverse roles in liver diseases. *Hepatology* 55 622–633. 10.1002/hep.25497 22120903 PMC3417333

[B35] Oliva J. Bardag-Gorce F. French B. A. Li J. French S. W. (2009). The regulation of non-coding RNA expression in the liver of mice fed DDC. *Exp. Mol. Pathol.* 87 12–19. 10.1016/j.yexmp.2009.03.006 19362547 PMC2885145

[B36] Orchard S. Ammari M. Aranda B. Breuza L. Briganti L. Broackes-Carter F. (2014). The MIntAct project—IntAct as a common curation platform for 11 molecular interaction databases. *Nucleic Acids Res.* 42 D358–D363. 10.1093/nar/gkt1115 24234451 PMC3965093

[B37] Piñero J. Bravo À. Queralt-Rosinach N. Gutiérrez-Sacristán A. Deu-Pons J. Centeno E. (2017). DisGeNET: a comprehensive platform integrating information on human disease-associated genes and variants. *Nucleic Acids Res.* 45 D833–D839. 10.1093/nar/gkw943 27924018 PMC5210640

[B38] Piñero J. Gonzalez-Perez A. Guney E. Aguirre-Plans J. Sanz F. Oliva B. (2018). Network, transcriptomic and genomic features differentiate genes relevant for drug response. *Front. Genet.* 9:412. 10.3389/fgene.2018.00412 30319692 PMC6168038

[B39] Rouillard A. D. Gundersen G. W. Fernandez N. F. Wang Z. Monteiro C. D. McDermott M. G. (2016). The harmonizome: a collection of processed datasets gathered to serve and mine knowledge about genes and proteins. *Database* 2016:baw100. 10.1093/database/baw100 27374120 PMC4930834

[B40] Schubert M. Klinger B. Klünemann M. Sieber A. Uhlitz F. Sauer S. (2018). Perturbation-response genes reveal signaling footprints in cancer gene expression. *Nat. Commun.* 9:20. 10.1038/s41467-017-02391-6 29295995 PMC5750219

[B41] Schueller F. Roy S. Vucur M. Trautwein C. Luedde T. Roderburg C. (2018). The role of miRNAs in the pathophysiology of liver diseases and toxicity. *Int. J. Mol. Sci.* 19:E261. 10.3390/ijms19010261 29337905 PMC5796207

[B42] Schwabe R. F. Brenner D. A. (2006). Mechanisms of liver injury. I. TNF-α-induced liver injury: role of IKK, JNK, and ROS pathways. *Am. J. Physiol. Liver Physiol.* 290 G583–G589. 10.1152/ajpgi.00422.2005 16537970

[B43] Seki E. Brenner D. A. Karin M. (2012). A liver full of JNK: signaling in regulation of cell function and disease pathogenesis, and clinical approaches. *Gastroenterology* 143 307–320. 10.1053/j.gastro.2012.06.004 22705006 PMC3523093

[B44] Souza T. Jennen D. van Delft J. van Herwijnen M. Kyrtoupolos S. Kleinjans J. (2016). New insights into BaP-induced toxicity: role of major metabolites in transcriptomics and contribution to hepatocarcinogenesis. *Arch. Toxicol.* 90 1449–1458. 10.1007/s00204-015-1572-z 26238291 PMC4873527

[B45] Souza T. M. van den Beucken T. Kleinjans J. C. S. Jennen D. G. J. (2017). Inferring transcription factor activity from microarray data reveals novel targets for toxicological investigations. *Toxicology* 389 101–107. 10.1016/J.TOX.2017.07.008 28743512

[B46] Sutherland J. J. Jolly R. A. Goldstein K. M. Stevens J. L. (2016). Assessing concordance of drug-induced transcriptional response in rodent liver and cultured hepatocytes. *PLoS Comput. Biol.* 12:e1004847. 10.1371/journal.pcbi.1004847 27028627 PMC4814051

[B47] Sutherland J. J. Webster Y. W. Willy J. A. Searfoss G. H. Goldstein K. M. Irizarry A. R. (2017). Toxicogenomic module associations with pathogenesis: a network-based approach to understanding drug toxicity. *Pharmacogenomics J.* 18 377–390. 10.1038/tpj.2017.17 28440344

[B48] Tacke F. Luedde T. Trautwein C. (2009). Inflammatory pathways in liver homeostasis and liver injury. *Clin. Rev. Allergy Immunol.* 36 4–12. 10.1007/s12016-008-8091-0 18600481

[B49] Takahashi K. Yan I. Haga H. Patel T. (2014). Long noncoding RNA in liver diseases. *Hepatology* 60 744–753. 10.1002/hep.27043 24493213 PMC4110118

[B50] Tarca A. L. Draghici S. Khatri P. Hassan S. S. Mittal P. Kim J. (2009). A novel signaling pathway impact analysis. *Bioinformatics* 25 75–82. 10.1093/bioinformatics/btn577 18990722 PMC2732297

[B51] Tawa G. J. AbdulHameed M. D. M. Yu X. Kumar K. Ippolito D. L. Lewis J. A. (2014). Characterization of chemically induced liver injuries using gene co-expression modules. *PLoS One* 9:e107230. 10.1371/journal.pone.0107230 25226513 PMC4165895

[B52] Teng K.-Y. Ghoshal K. (2015). Role of noncoding RNAs as biomarker and therapeutic targets for liver fibrosis. *Gene Expr.* 16 155–162. 10.3727/105221615X14399878166078 26637395 PMC4689200

[B53] The Gene Ontology Consortium (2017). Expansion of the gene ontology knowledgebase and resources. *Nucleic Acids Res.* 45 D331–D338. 10.1093/nar/gkw1108 27899567 PMC5210579

[B54] Türei D. Korcsmáros T. Saez-Rodriguez J. (2016). OmniPath: guidelines and gateway for literature-curated signaling pathway resources. *Nat. Methods* 13 966–967. 10.1038/nmeth.4077 27898060

[B55] van Dam S. Võsa U. van der Graaf A. Franke L. de Magalhães J. P. (2017). Gene co-expression analysis for functional classification and gene–disease predictions. *Brief. Bioinform.* 19 575–592. 10.1093/bib/bbw139 28077403 PMC6054162

[B56] Wang X. Thijssen B. Yu H. (2013). Target essentiality and centrality characterize drug side effects. *PLoS Comput. Biol.* 9:e1003119. 10.1371/journal.pcbi.1003119 23874169 PMC3708859

[B57] Wei C.-H. Kao H.-Y. Lu Z. (2013). PubTator: a web-based text mining tool for assisting biocuration. *Nucleic Acids Res.* 41 W518–W522. 10.1093/nar/gkt441 23703206 PMC3692066

[B58] Xu Y. Wu J. Peng X. Yang T. Liu M. Chen L. (2017). LncRNA LINC00341 mediates PM 2.5 -induced cell cycle arrest in human bronchial epithelial cells. *Toxicol. Lett.* 276 1–10. 10.1016/j.toxlet.2017.03.026 28366736

[B59] Yıldırım M. A. Goh K.-I. Cusick M. E. Barabási A.-L. Vidal M. (2007). Drug—target network. *Nat. Biotechnol.* 25 1119–1126. 10.1038/nbt1338 17921997

[B60] Zhang J. D. Berntenis N. Roth A. Ebeling M. (2014). Data mining reveals a network of early-response genes as a consensus signature of drug-induced *in vitro* and *in vivo* toxicity. *Pharmacogenomics J.* 14 208–216. 10.1038/tpj.2013.39 24217556 PMC4034126

[B61] Zhang Y. Xu N. Xu J. Kong B. Copple B. Guo G. L. (2014). E2F1 is a novel fibrogenic gene that regulates cholestatic liver fibrosis through the Egr-1/SHP/EID1 network. *Hepatology* 60 919–930. 10.1002/hep.27121 24619556 PMC4146672

[B62] Zhao J. Liu Y. Wei X. Yuan C. Yuan X. Xiao X. (2009). A novel WD-40 repeat protein WDR26 suppresses H_2_O_2_-induced cell death in neural cells. *Neurosci. Lett.* 460 66–71. 10.1016/j.neulet.2009.05.024 19446606

[B63] Zhou Z. Liu H. Wang C. Lu Q. Huang Q. Zheng C. (2015). Long non-coding RNAs as novel expression signatures modulate DNA damage and repair in cadmium toxicology. *Sci. Rep.* 5:15293. 10.1038/srep15293 26472689 PMC4607885

[B64] Zhu Q. Luo Z. Lu G. Gui F. Wu J. Li F. (2018). LncRNA FABP5P3/miR-589-5p/ZMYND19 axis contributes to hepatocellular carcinoma cell proliferation, migration and invasion. *Biochem. Biophys. Res. Commun.* 498 551–558. 10.1016/j.bbrc.2018.03.017 29522715

