# Supplementary material for: Embracing the Dark Side: Computational Approaches to Unveil the Functionality of Genes Lacking Biological Annotation in Drug-Induced Liver Injury
Source: Front Genet. 2018 Nov 20;9:527. doi: 10.3389/fgene.2018.00527 (PMC6255978; doi:10.3389/fgene.2018.00527)
Supplement: DATA SHEET S1 — Supplementary Figures. [file Data_Sheet_1.PDF]

Supplementary Data 2

Embracing the dark side: computational approaches to unveil the functionality of genes lacking biological annotation in drug-induced liver injury

Terezinha Souza, Panuwat Trairatphisan, Janet Piñero, Laura Furlong, Julio Saez-Rodriguez, Jos Kleinjans, Danyel Jennen

Table of Contents

Figure 1. Venn Diagrams of the overlap of DEGs across compounds from DILI risk groups analyzed **2**

Figure 2. Summary of genes mapped to database resources. Note: 4210 genes from the Affymetrix chip (tagged as "array" in the graph) were not found in any of the databases used. **3**

Figure 3. Venn diagram containing the dark gene coverage in 4 different human protein-protein interaction databases. **4**

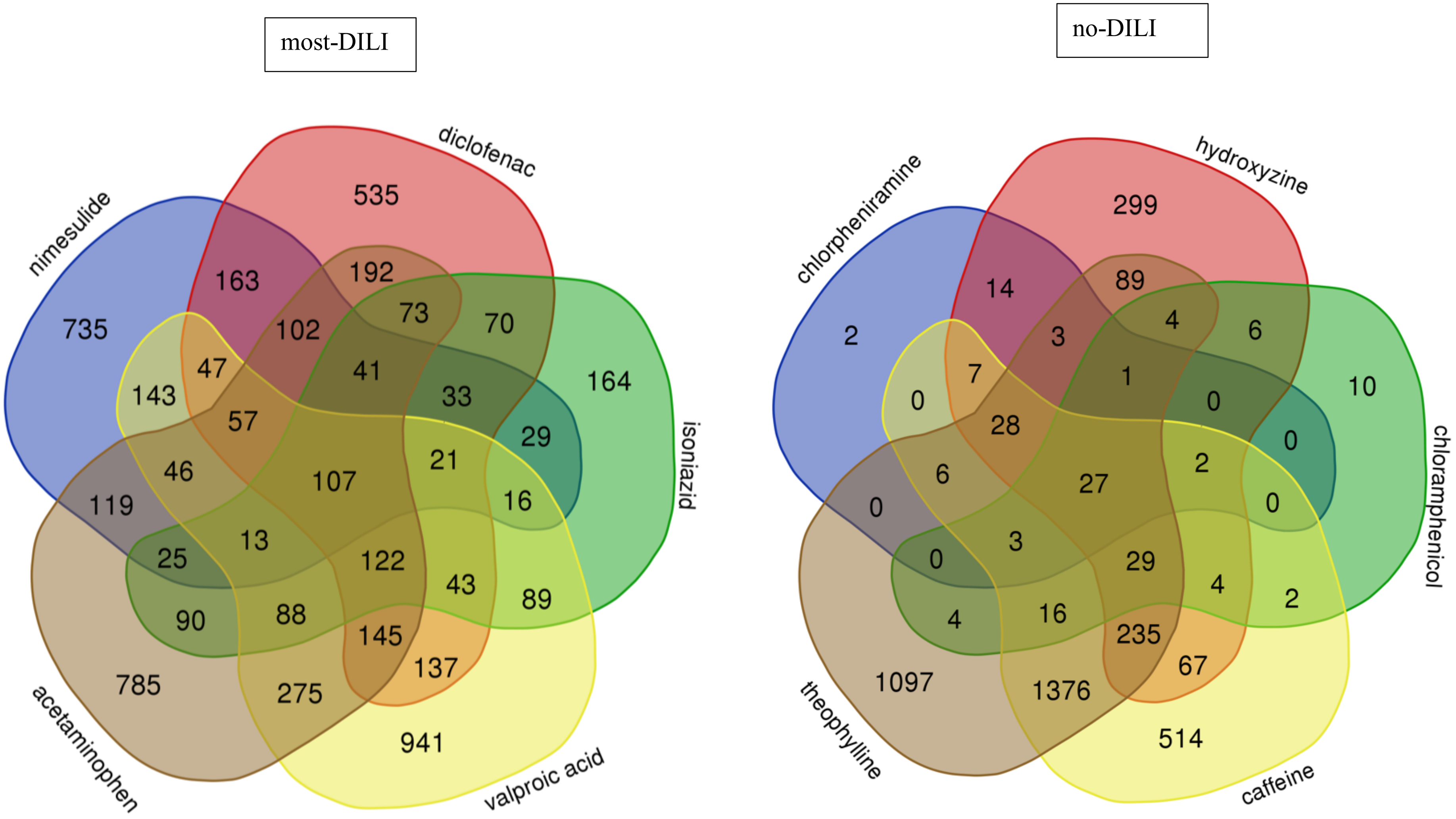

Figure 1. Venn Diagrams of the overlap of DEGs across compounds from DILI risk groups analyzed

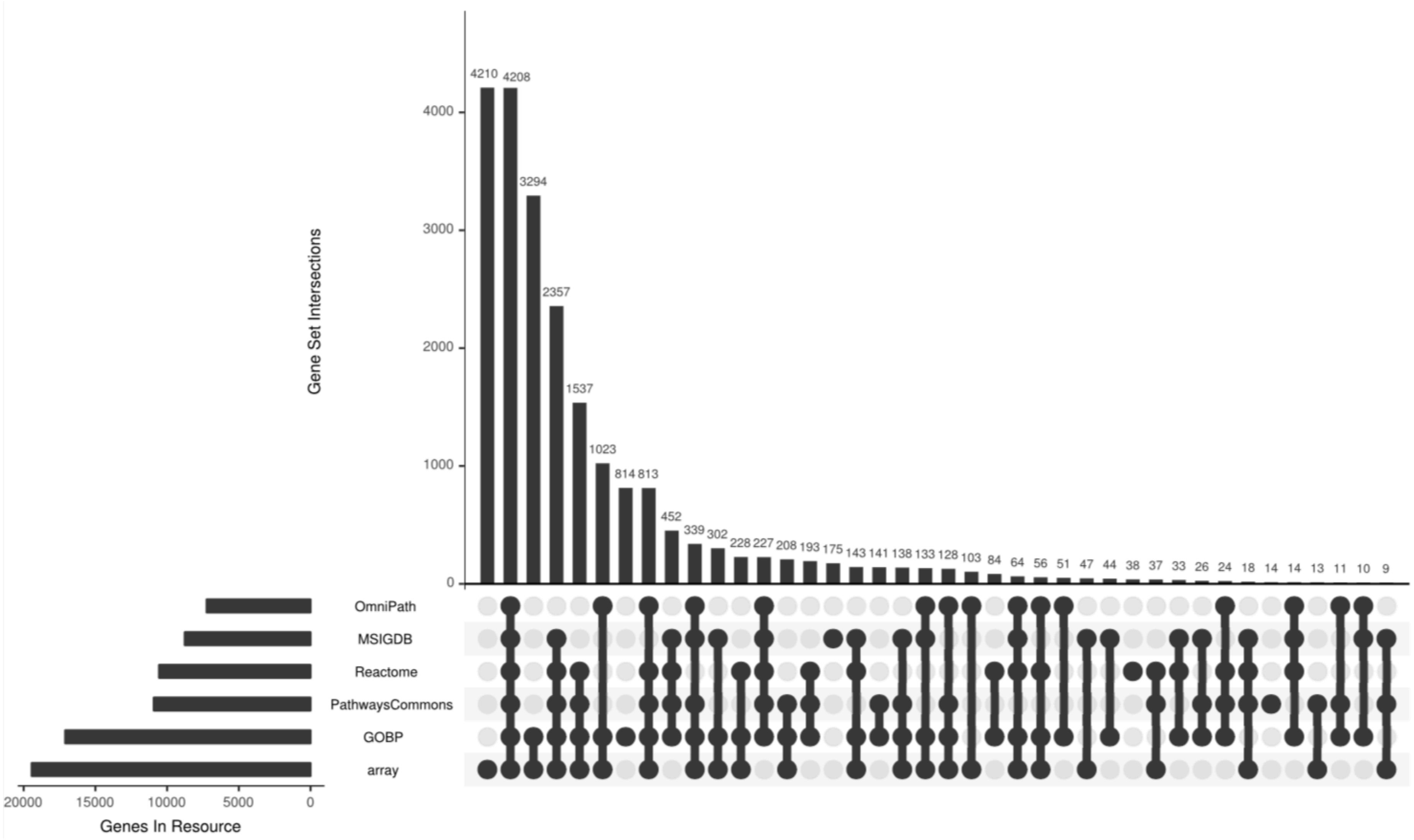

Figure 2. Summary of genes mapped to database resources. Note: 4210 genes from the Affymetrix chip (tagged as "array" in the graph) were not found in any of the databases used.

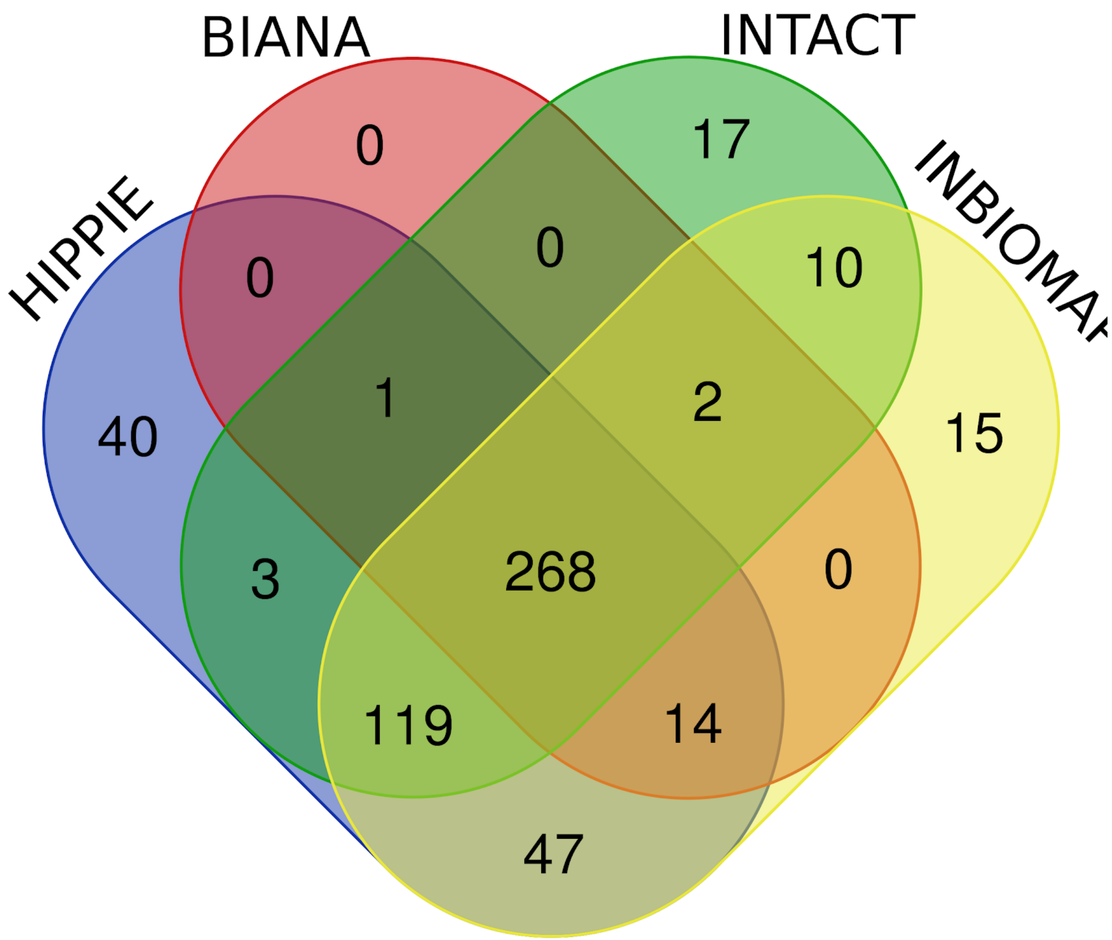

Figure 3. Venn diagram containing the dark gene coverage in 4 different human protein-protein interaction databases.
