# Supplementary material for: Embracing the Dark Side: Computational Approaches to Unveil the Functionality of Genes Lacking Biological Annotation in Drug-Induced Liver Injury
Source: Front Genet. 2018 Nov 20;9:527. doi: 10.3389/fgene.2018.00527 (PMC6255978; doi:10.3389/fgene.2018.00527)
Supplement: DATA SHEETS S2 and S3 — Scripts (.R) used in all analyses conducted in this study as well as mapping to databases and IDs. [file Data_Sheet_2.ZIP › Suppl.Data3_P.1/ensembl2entrezid_data/README.docx]

The file mart_exportgenetypes.txt was downloaded via ensembl biomart on January 4

The query:

http://www.ensembl.org/biomart/martview/af61ad29d7b8ea1bb6e64468725ea060?VIRTUALSCHEMANAME=default&ATTRIBUTES=hsapiens_gene_ensembl.default.feature_page.ensembl_gene_id|hsapiens_gene_ensembl.default.feature_page.ensembl_transcript_id&FILTERS=&VISIBLEPANEL=resultspanel
